# The cold - resistance mechanism of a mutagenic *Volvariella volvacea* strain VH3 with outstanding traits revealed by transcriptome profiling

**DOI:** 10.1186/s12866-021-02396-8

**Published:** 2021-12-08

**Authors:** Peng Li, Cong Hu, Yujie Li, Lei Ge, Guogan Wu, Beibei Lv, Wei Jiang, Dandan Xi

**Affiliations:** 1 Shanghai Key Laboratory of Agricultural Genetics and Breeding, Biotechnology Research Institute, Shanghai Academy of Agricultural Sciences, Shanghai, 201106 China; 2 Shanghai Key Laboratory of Protected Horticultural Technology, Protected Horticultural Research Institute, Shanghai Academy of Agricultural Sciences, Shanghai, 201106 China

**Keywords:** Straw mushroom, *Volvariella volvacea*, Cold, VH3

## Abstract

**Background:**

The straw mushroom (*Volvariella volvacea*) is one of the important vegetables that is popular for its delicious taste. However, the straw mushroom is sensitive to low temperature, resulting in economic loss during transportation and storage. We obtained a novel straw mushroom strain, named VH3, via ultraviolet mutagenesis.

**Results:**

Our study revealed that VH3 exhibited high cold resistance compared to an ordinary straw mushroom cultivar, V23. We found that the electrolyte leakages of VH3 were always significantly lower than that of V23 treated with 4 °C for 0 h, 2 h,4 h, 8 h, 16 h, and 24 h. Before cold treatment (0 h), there were no difference of MDA contents, SOD activities, and CAT activities between VH3 and V23. At the late stage (8 h, 26 h, and 24 h) of cold treatment, the MDA contents of VH3 were lower while both the SOD and CAT activities were higher than those of V23. To investigate the potential mechanisms of VH3 cold resistance, we performed transcriptome sequencing to detect the transcriptome profiling of VH3 and V23 after 0 h and 4 h cold treatment. Transcriptome sequencing revealed that 111 differentially expressed genes (DEG) between V23 (0 h) and VH3 (0 h) (V23–0_vs_VH3–0), consisting 50 up-regulated and 61 down-regulated DEGs. A total of 117 DEGs were obtained between V23 (4 h) and VH3(4 h) (V23–4_vs_VH3–4), containing 94 up-regulated and 23 down-regulated DEGs. Among these DEGs, VVO_00021 and VVO_00017 were up-regulated while VVO_00003, VVO_00004, VVO_00010, and VVO_00030 were down-regulated in V23–0_vs_VH3–0 and VH3–4_vs_V23–4. KEGG and GO analysis revealed that the 6 DEGs were annotated to pathways related to cold stress. Besides, the GA3 content was also decreased in VH3.

**Conclusions:**

Collectively, our study first revealed that the increased cold resistance of VH3 might be caused by the expression change of VVO_00003, VVO_00004, VVO_00017, VVO_00021, and VVO_00030, and decreased GA_3_.

**Supplementary Information:**

The online version contains supplementary material available at 10.1186/s12866-021-02396-8.

## Introduction

Straw mushroom (*Volvariella volvacea* (Bull.exFr) Sing) is a kind of high temperature edible fungus which grows in tropical and subtropical areas, belonging to *Plutaceae* family. Straw mushroom benefits of human health, including prevention of cancer and scurvy, and decreasing blood pressure and cholesterol levels. Besides, it is also rich in amino acid, vitamin C1, vitamin B1, and vitamin PP [1]. Therefore, straw mushroom becomes one of the most popular mushroom, of which the planting area increases year after year.

Straw mushroom is a subtropical-edible fungus that is sensitive to temperature changes. Studies have revealed that the suitable temperature for straw mushroom mycelium is 30-35 °C. The mycelium will grow slowly at the temperature lower than 15 °C. When temperature is lower than 4 °C, cryogenic autolysis of fruiting body will occur. Weight loss rate, relative electric conductivity, malondialdehyde (MDA) content, and neutral protease will increase, resulting in mycelium soft and even death [2]. This characteristic seriously restricts the growth, storage, and transportation of straw mushroom.

Generally, a method to increase yield of mushroom is to improve the low-temperature -resistance of straw mushroom. However, the molecular mechanism of straw mushroom in response to low temperature is rarely studied. Based on the published whole genome of straw mushroom, 11,097 protein-encoding genes were predicted, of which 5516 genes could be annotated [3]. Benefiting from published genome, it was found 55 down-regulated and 25 up-regulated differentially expressed genes (DEGs) in response to 4 °C treatment subsequently [4]. Among the up-regulated DEGs, VVO_05539 encodes a putative protein with an F-box domain whereas VVO_00268 encodes a putative protein containing a conserved domain related to ubiquitin-conjugating enzyme E2. A senescence-associated protein and a His-Asn-His nucleases associated protein, encoded by VVO_05394 and VVO_06566, respectively, are also identified. Among down-regulated DEGs, 8 genes encode putative proteins related to heat shock proteins (HSP), which have been proved to be associated with low temperature in *Saccharomyces cerevisiae*. A small GTPase, encoded by *vvran1*, was induced by cold stress and hydrogen peroxide stress [5]. A latest research revealed that cold stress could decrease protein translation through ubiquitination of ribosomal proteins in mycelium [6].UBE2(E2), a type of ubiquitin-conjugating enzyme, was upregulated after cold stress and bound to SSB2, a ribosome-associated molecular chaperone. Conversely, UBE2 inhibitor could increase the cold stress resistance of *V.volvacea* [6].

To investigate the molecular mechanism of high cold resistance of VH3, our study performed transcriptome sequencing and obtained 2 up-regulated DEGs, VVO_00021 and VVO_00017, and 4 down-regulated genes, VVO_00003, VVO_00004, VVO_00010, and VVO_00030. The VVO_00003, VVO_00004, VVO_00021, VVO_00017, and VVO_00030 were annotated to stress related pathways according to KEGG and GO analysis. We also found VH3 biosynthesized less GA_3_ compared to V23. Therefore, we considered that the high cold resistance of VH3 might be resulted from the changes of gene expression levels and less GA_3_ content.

## Results

**Fig. 1:**
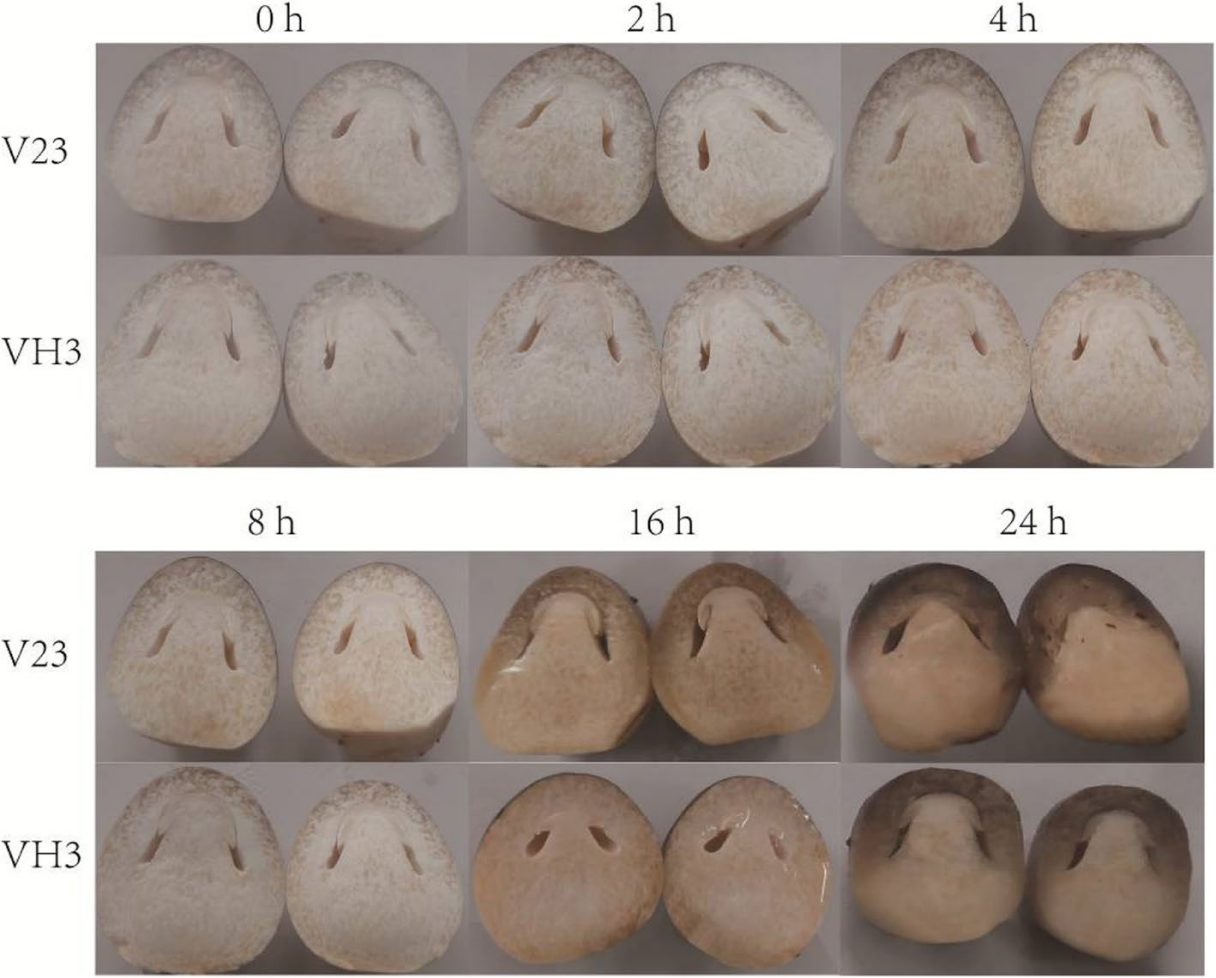
Morphology changes of straw mushroom in response to cold stress. The fruiting body of straw mushroom was exposed to cold stress (4 °C) for 0 h, 2 h, 4 h, 8 h, 16 h, and 24 h

To study the resistance of V23 and VH3 to cold stress, V23 and VH3 were exposed to cold conditions (4 °C) for 0 h, 2 h, 4 h, 8 h, 16 h, and 24 h. Results showed that the morphology VH3 did not change significantly with 2 h, 4 h, and 8 h cold stress treatment (CST) compared with V23 (Fig. 1). At 16 h CST, water droplets appeared in the surface of straw mushroom, and biofilm turned brown both in V23 and VH3. However, V23 started to cryogenic autolysis while VH3 did not after 16 h cold stress (Fig. 1). At 24 h treatment, cryogenic autolysis was observed in V23 and VH3 (Fig. 1). Thus, VH3 showed higher resistance to cold stress than V23.

**Fig. 2:**
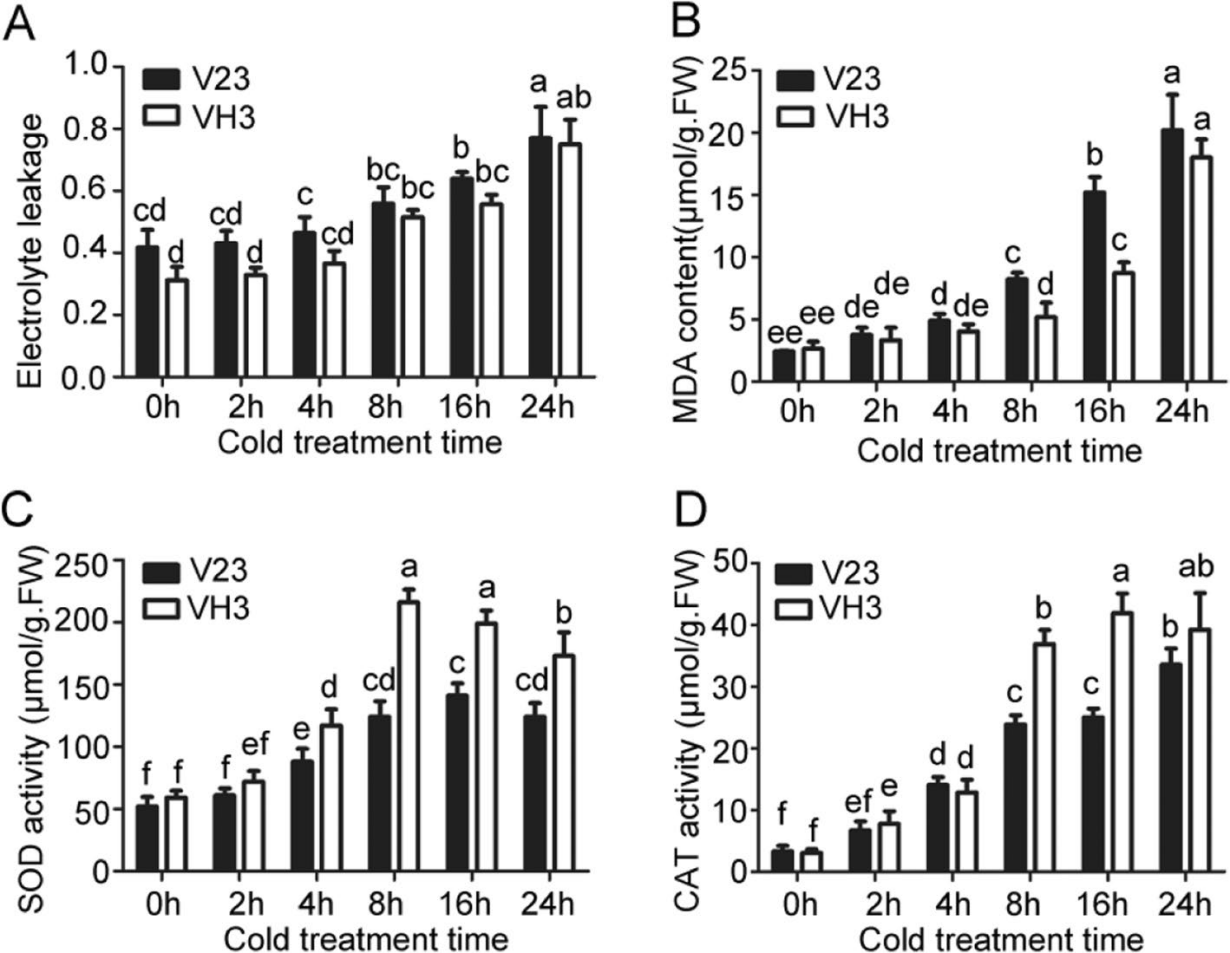
Effect of cold stress on *V.volvacea* physiology. Electrolyte leakage (**A**), MDA contents (**B**), SOD activities(**C**), and CAT activities (**D**) in V23 and VH3 were evaluated. Data shown represent average ± standard error with three replications. Different letters represent significance (*P* < 0.05)

When suffered from stresses, cell membrane will be injured, changing the structure and increasing electrolyte leakage and malondialdehyde (MDA) contents. Besides, to response to stresses, fungus will also produce antioxidant enzymes to eliminate reactive oxygen species (ROS), byproducts of aerobic metabolism and toxic to organisms. Therefore, we first analyzed electrolyte leakage and MDA contents. Results revealed that electrolyte leakage, MDA content, superoxide dismutase (SOD) activity, and catalase (CAT) activity were increased with CST time (Fig. 2). The electrolyte leakage in VH3 was always lower than that in V23 before and after CST (Fig. 2A). At 0 h and 2 h CST, MDA contents in VH3 exhibited no significant difference with that in V23 and then started to lower than that in V23 after 4 h CST (Fig. 2B). Contrary, both SOD and CAT, used for scavenging H_2_O_2_, were significantly increased in VH3 compared to those in V23 after 2 h CST (Fig. 2C and D). These data showed that VH3 increased cold resistance resulted from decreased electrolyte leakage and MDA contents and increased SOD and CAT activities to scavenge excessive H_2_O_2_.

### Transcriptome analysis

To further discover the molecular mechanism of VH3 resistance to cold stress, the total RNA from fruiting body of VH3 and V23 at 0 h and 4 h CST was used for transcriptome sequencing. Then, after filtering low quality reads, we obtained 156,528,742, 138,116,660,103,088,102, and 160,960,664 reads for V23 (0 h), VH3 (0H), V23 (4 h), and VH3(4 h), respectively (Table 1). Mapping ratio of each sample was above 90% (Table 1). The principal component analysis (PCA) showed that three biological repetitions cluster together (Sup Fig. 1). These data revealed that our transcriptome sequencing was reliable and could be used for further analysis.

**Table 1:** Summary for the *V.volvacea* transcriptome sequencing

Sample ID	All reads	Mapped reads	Mapped unique reads	Mapped multi reads	Mapping ratio
V23–1-0 h	55,238,836	53,265,984	53,103,471	162,513	96.4%
V23–2-0 h	42,739,172	40,895,426	40,772,061	123,365	95.7%
V23–3-0 h	58,550,734	56,463,853	56,269,951	193,902	96.4%
VH3–1-0 h	43,594,366	41,983,595	41,868,586	115,009	96.3%
VH3–2-0 h	47,533,982	45,752,561	45,623,281	129,280	96.3%
VH3–3-0 h	46,988,312	45,264,508	45,129,324	135,184	96.3%
V23–1-4 h	30,034,450	28,452,407	28,368,106	84,301	94.7%
V23–2-4 h	34,169,678	32,247,439	32,147,455	99,987	94.4%
V23–3-4 h	38,880,558	37,189,663	37,084,684	104,979	95.7%
VH3–1-4 h	50,335,294	48,275,125	48,130,514	144,611	95.9%
VH3–2-4 h	29,338,194	27,894,844	27,819,630	75,214	95.1%
VH3–3-4 h	81,287,176	77,750,356	77,523,960	226,396	95.6%

**Fig. 3:**
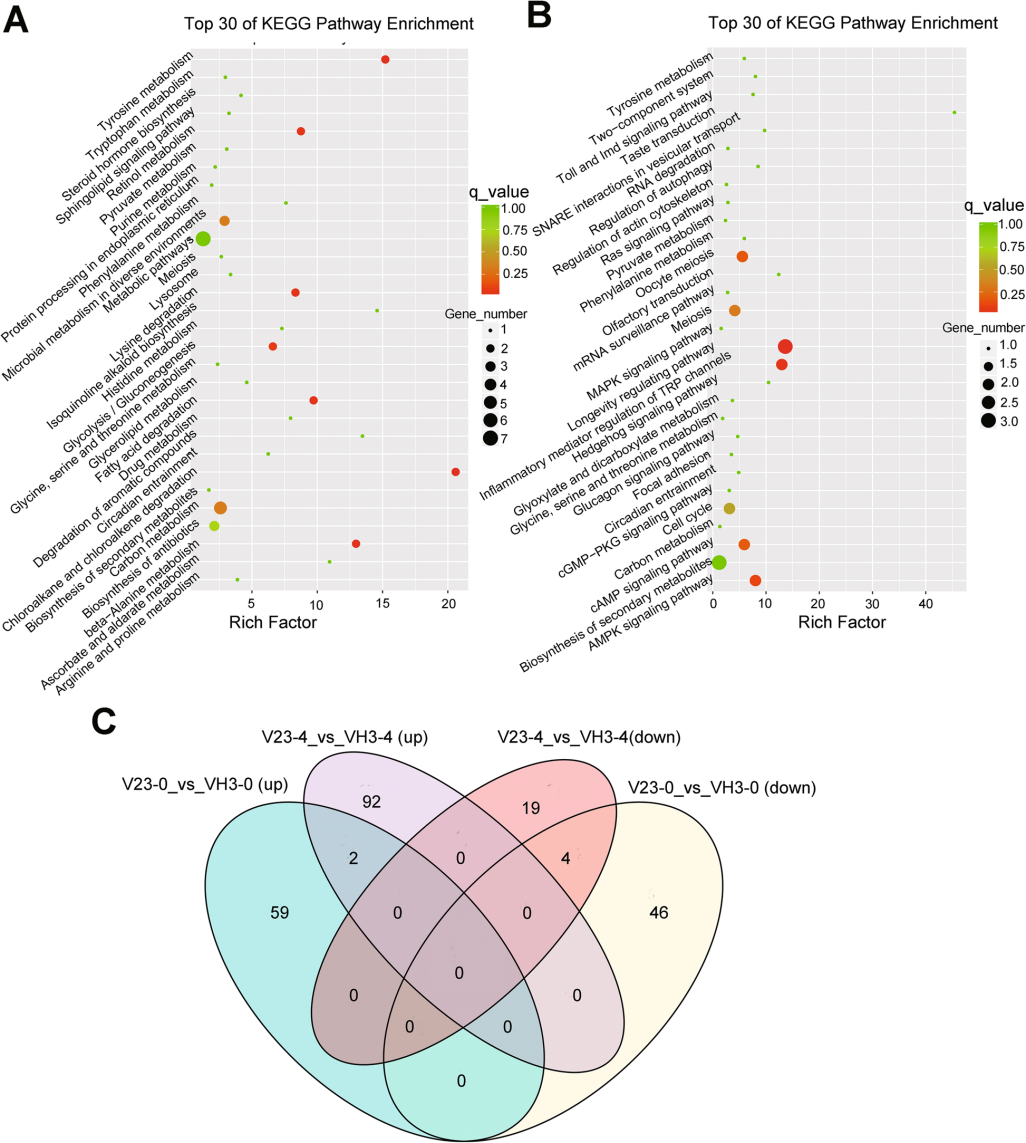
KEGG enrichment and Venn analysis. Top 30 of KEGG pathways of DEGs in V23–0_vs_VH3–0 (**A**) and V23–4_vs_VH3–4 (**B**). The rich factor (X axis) represents the ratio of DEGs to the total genes in the same pathway. The larger the Rich factor, the greater the enrichment. The larger the point, the greater the number of DEGs enriched in the pathway. A high q-value (adjusted *p*-value) is represented by green and a low q-value is represented by red (q < 0.05). The redder the color of the dots, the more significant the enrichment. (C) Venn analysis of DEGs between V23 and VH3 at 0 h and 4H cold stress treatment (CST)

Comparisons of gene expression levels between V23 and VH3 at 0 h and 4 h (V23–0_vs_VH3–0, V23–4_vs_VH3–4) were determined by fold change > = 2 and FDR < 0.001. Then we totally found 111 differentially expressed genes (DEGs) in V23–0_vs_VH3–0 and 117 DEGs in V23–4_vs_VH3–4 (Sup Table 1). To further study gene functions and pathways, kyoto encyclopedia of genes and genome (KEGG) and gene ontology (GO) analysis were performed. In comparison of V23–0_vs_VH3–0, KEGG pathway analysis showed that 7 DEGs were annotated to “Metabolic pathways”, 5 to “Biosynthesis of secondary metabolites”, and 3 to each pathway of “ Microbial metabolism in diverse environments” and “ Biosynthesis antibiotics” (Fig. 3A). In V23–4_vs_VH3–4, 3 DEGs were annotated to each pathway of “Longevity regulating pathway” and “Biosynthesis of secondary metabolites”(Fig. 3B). Two DEGs were annotated to each of “Inflammatory mediator regulation of TRP channels”, “AMPK signaling pathway”,“cAMP signaling pathway”, “Oocyte meiosis”,” Meiosis”, and “ Cell cycle” (Fig. 3B).

A Venn analysis diagram showed that 2 DEGs were both up-regulated at 0 h and 4 h while 4 DEGs were down-regulated at 0 h and 4 h (Fig. 3C). Two up-regulated genes were encoded byVVO_00021 and VVO_00017. VVO_00021was annotated to GO terms, like “ MAP kinase activity”,“ MAP kinase kinase activity”, and “ calcium-dependent protein kinase C activity” while VVO_00017 was annotated to the KEGG pathway “ Biosynthesis of secondary metabolites”. MAPK cascades, and calcium-dependent protein kinase were identified to regulate cold stress through modulating ICE1 protein stability in plants [7, 8]. Secondary metabolites play important roles during cold stress in plants and fungi [9, 10]. Four down-regulated transcripts were encoded by VVO_00003, VVO_00004, VVO_00010, and VVO_00030, respectively. VVO_00003 encoded a ganoderma gungal immunomodulatory protein while VVO_00004 encoded an immunomodulatory protein Ling zhi-8. Both VVO_00003 and VVO_00004 were annotated to the GO term of regulation of immune system process. VVO_00030 encoded a putative protein that contains an acetyl xylan esterase II region and was annotated to the GO term of Metabolic process. Xylan is one of the hemicellulose, a major component of plant cell walls, can be degraded by xylanases into xylose, thus breaking down the cell walls [11, 12]. Xylan esterases remove the O-acetyl groups from the xylose residues, assisting xylanases to degrade xylan [13]. These data suggested that the high cold-resistance of VH3 might be resulted from up-regulated VVO_00021 and VVO_00017 and down-regulated VVO_00003, VVO_00004, and VVO_00030 expression levels.

### Verification of transcriptome sequencing by qRT-PCR

To verify the results of transcriptome sequencing, a qRT-PCR analysis was performed.

**Fig. 4:**
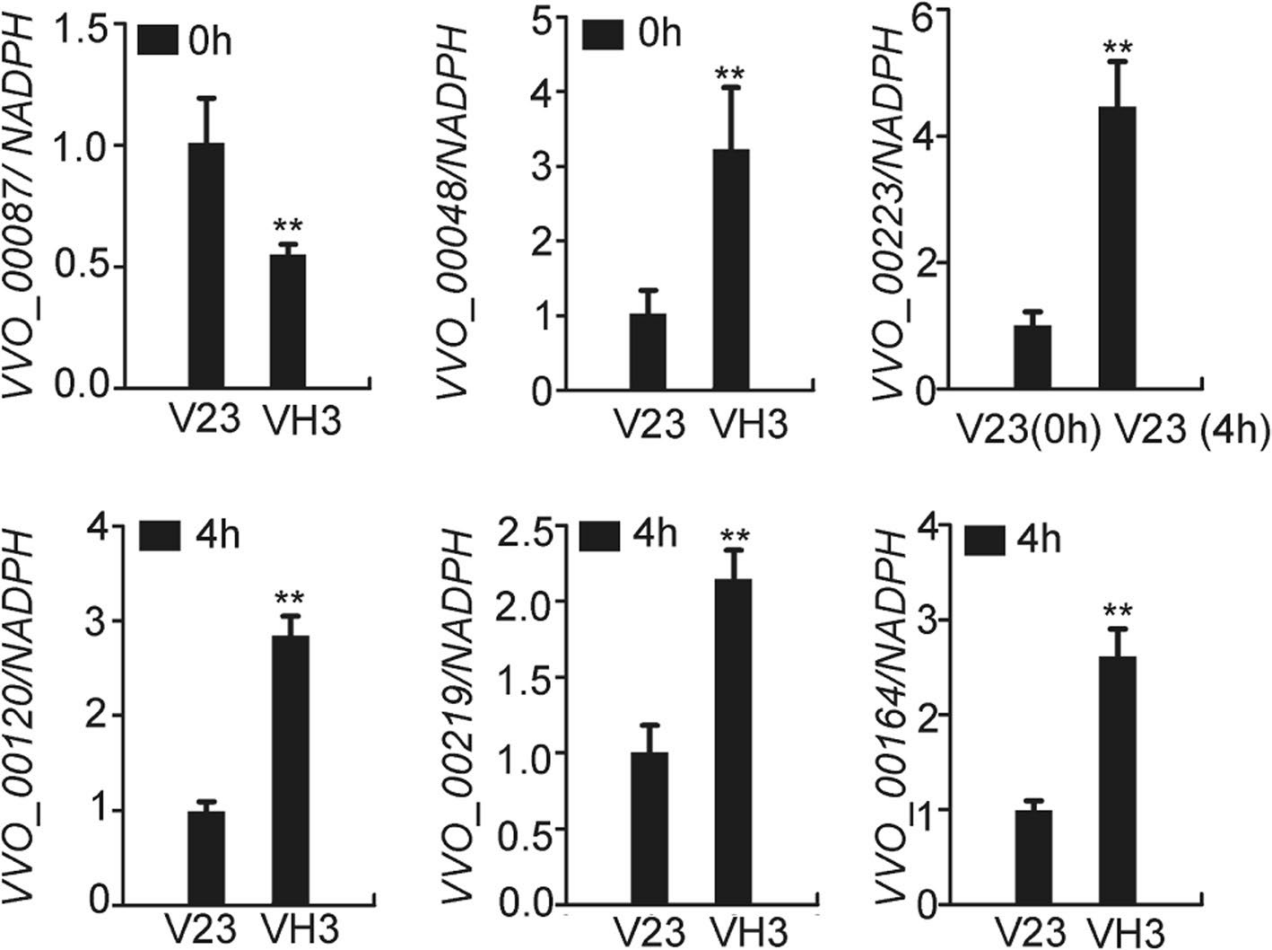
Relative gene expression levels. Gene expression levels were calculated between V23 and VH3 after 0 h and 4 h CST. Data shown are average ± standard error with three replications. ** *P* < 0.01(Students’ *t*-test)

Results showed that VVO_0087 was down-regulated in VH3 while VVO_00048 was up-regulated in VH3 at 0 h CST (Fig. 4). At 4 h CST, 3 genes, VVO_00120, VVO_00219, and VVO_00164, were significantly up-regulated in VH3 (Fig. 4). Additionally, the expression of VVO_00223 was also compared between V23–0 and V23–4 and up-regulated in V23 after 4 h CST (Fig. 4). These gene expression change was consistent with those in transcriptome sequencing results.

## Discussion

*V.volvacea* is an essential edible fungus with high nutrition and sensitive to temperature changes. Routine storage temperatures (4 °C) will cause *V.volvacea* cryogenic autolysis and great economic loss. To solve this issue of cryogenic autolysis, lots of efforts have been made. Previous studies reported that chilling stress inhibited protein translation and degradation [6]. Besides, an ubiquitin-conjugating enzyme E2, UBE2, was up-regulated and affected ubiquitination after cold stress in *V.volvacea* [6]. One of small GTPasse, *Vvran1*, was induce by cold stress and hydrogen peroxide stress and repressed by an NADPH oxidase-specific inhibitor [5]. In our study, we found 5 DEGs involved in cold stress by transcriptome sequencing, 2 up-regulated genes (VVO_00021 and VVO_00017) and 4 down-regulated genes (VVO_00003, VVO_00004, VVO_000010, and VVO_00030).

Cell wall protects cells and provides nutrients when cells suffer stresses. To response to cold stress, cell walls thicken with changed contents of cell wall sugars. Our data observed a new gene, VVO_00030, encoding an acetyl xylan esterase II. Down-regulated VVO_00030 might reduce the degradation of xylan and protect cell walls of VH3, thus increasing the cold resistance of VH3. VVO_00004, encoding a Ling Zhi-8 domain. Ling Zhi-8 is purified from *Ganoderma lucidium* and acts as an immunomodulatory protein. Most studies focused on the roles of Ling Zhi-8 in human diseases, like ameliorates nonalcoholic fatty liver, early atherogenesis, and lung cancer [14, 15]. Our study first observed the down expression of VVO_00004 in VH3 after 0 h and 4 h CST, indicating VVO_00004 may participate in cold stress.

MDA is a byproduct of membrane lipid peroxidation and represents the damage of ROS to membrane. SOD and CAT are two key antioxidant enzymes involved in ROS scavenging and protecting membrane against peroxidation. In our study, increased SOD and CAT activities after 2 h CST suggests the decreased ROS contents in VH3, which is consistent with the decreased MDA contents in VH3 (Fig. 2). Our data also reported a new gene, VVO_00017, encoding a copper-containing primary amine oxidase. Copper amine oxidases catalyze primary amines oxidation and produce hydrogen peroxide which is further converted into water by CAT [16, 17]. In our study, both VVO_00017 expression and CAT activities were increased in VH3 (Fig. 2 and Fig. 3), suggesting more primary amines were degraded and more hydrogen peroxide was converted into water, resulting in high resistance of VH3.

Abscisic acid (ABA) and gibberellic acid (GA) are two hormones mediate thermotolerance in plants. ABA application was reported to increase cold resistance in Bermudagrass and maize [18, 19]. Meanwhile, cold stress reduces the production of bioactive GA and promotes DELLA accumulation in *Arabidopsis* [20]. In tomato, lower temperature could increase bioactive GA_1_ and GA_4_ contents and GA related genes [21]. Besides, a series studies found that ABA can be also produced by a small number of fungi, including *Botrytis cinerea* and *Cercospora pini-densiflorae* [22, 23]. The GA was first identified in rice pathogenic fungus and biosynthesized in *Fusarium* species and *Paecilomyces* sp. ZB [24, 25]. These data suggest us that ABA and GA might be responsible for the increased cold resistance of VH3. To further study whether hormones contribute to the cold resistance of VH3, our study first detected the two hormones in edible fungi and found GA_3_ content was significantly decreased in VH3 while the contents of ABA, GA_1_, GA_4_, and GA_7_ cannot be detected in V23 and VH3. (Sup Fig. 2). The decreased GA_3_ contents resulted in increased cold tolerance of VH3. However, we did not found DEGs annotated to GA biosynthesis or GA signal transduction pathways in comparison V23–0_vs_VH3–0. The reason for this phenomenon may result from the limitation of transcriptome sequencing. Thus, the mechanism of VH3 change GA_3_ content still remains unclear.

## Conclusions

Collectively, our study first reported that VVO_00021, VVO_00017, VVO_00003, VVO_00004, VVO_00010, and VVO_00030 were associated with cold stress in straw mushroom through transcriptome sequencing. Additionally, we found the GA_3_ content was decreased in VH3 which might be a reason for high cold resistance of VH3.

## Methods

### Materials

The *V.volvacea*, V23 and VH3, was supplied by Prof. Mingjie Chen (Institute of Edible Fungi, Shanghai Academy of Agricultural Sciences, China). VH3 has been identified to resistant to cold stress compared to V23 and deposited in Culture Collection Center of Shanghai Academy of Agricultural Sciences. The mycelia of the straw mushroom were incubated on a potato dextrose agar (PDA) plate (200 g/L potato, 20 g/L glucose, 20 g/L agar) for 3 days at 32 °C. Then mycelia were transferred to plate with 85% cottonseed coat, 10% wheat bran,4% lime, and 60% water content (pH 8–9) for 4–5 days at 32 °C. When mycelia were over the medium, the mycelia with the medium were incubated in cultivation medium until fruiting body grows.

### MDA content assay

To analyze the MDA content, 1 g fruiting body of fungus was ground with 10 ml trichloroacetic acid (TCA, 10 g/mL) followed by centrifuge. 2 mL thiobarbituric acid (TBA, 6 mg/mL) was added to 2 mL supernatant and then incubated in boiling water for 15 min. Following quickly cooling and centrifuge, harvested supernatant was used to test OD_450_, OD_532_, and OD_600_.

MDA content (μmol/L) =6.45 × (OD_532_- OD_600_)-0.56 × OD_450_.

### Antioxidant enzyme activity assays

The SOD activity was detected by the SOD test kit (www.cominbio.com). According to the instructions, 0.1 g fruiting body from straw mushroom were added with 1 mL extraction buffer and then homogenated on ice. Then supernatant after centrifugal were harvested. The reaction system was composed of 45 μL buffer 1, 2 μL buffer 2, 18 μL supernatant or water (negative control), 35 μL buffer3, and 100 μL buffer 4. Buffer 1–4 were supplied by the kit. The absorbance was detected at 560 nm by (TECAN INFINITE M PLEX).

*P* = [Absorbance (water)- Absorbance (sample)]/ Absorbance (water) × 100%.

SOD activity (U/g) = 111.1 × P/(1-P).

The CAT activity was also detected by the CAT test kit (www.cominbio.com). 1 mL extraction buffer was added to 0.1 g fruiting body and homogenated on ice. Supernatant was obtained after centrifugal. 10 μL supernatant was mixed with 190 μL working fluid to obtain the first absorbance (A1) at 240 nm by. After 1 min standing, the second absorbance (A2) was detected at 240 nm.

CAT activity (U/g) =918 × (A1-A2)/sample weight.

### Transcriptome sequence

The total RNA was isolated from V23 and VH3 with RNeasy mini kit (Qiagen, Germany). Paired-end libraries were synthesized by using the TruSeq® RNA Sample Preparation Kit (Illumina, USA) following TruSeq® RNA Sample Preparation Guide. Briefly, the poly-A containing mRNA molecules were purified by poly-T oligo-attached magnetic beads. Purified libraries were quantified by Qubit® 2.0Fluorometer (Life Technologies, USA) and validated by Agilent 2100 bioanalyzer (Agilent Technologies, USA) to confirm the insert size and calculate the mole concentration. Cluster was generated by cBot with the library diluted to 10 pM and then were sequenced on the Illumina HiSeq X-ten (Illumina, USA). The library construction and sequencing was performed at Shanghai Biotechnology Corporation.

Sequencing raw reads were preprocessed by filtering out rRNA reads, sequencing adapters, short-fragment reads (length < 25) and other low-quality reads (3′ base Q < 20) by Seqtk (https://github.com/lh3/seqtk). We used Hisat2(version:2.0.4) to map clean reads to straw mushroom genome [3]. After genome mapping, Stringtie (version:1.3.0) was run with a reference annotation to generate FPKM values for known gene models [26, 27]. Differentially expressed genes were identified using edgeR [28]. The *p*-value significance threshold in multiple tests was set by the false discovery rate (FDR) [29]. The fold-changes were also estimated according to the FPKM in each sample. The differentially expressed genes were selected using the following filter criteria: FDR ≤0.05 and fold-change ≥2. The differentially expressed genes were further analyzed with gene ontology (GO) and Kyoto Encyclopedia of Genes and Genomes (KEGG) analysis. The FDR correction was used (q-value< 0.05) to reduce false positive prediction of enriched KEGG pathways and GO terms.

### qRT-PCR analysis

Total RNA of straw mushroom was extracted by trizol and used as templates for cDNA synthesis by TaKaRa PrimeScript RT reagent Kit with gDNA Eraser (Takara, Japan) according to manufacturer’s instructions. Then, qRT-PCR was performed with SYBR Premix EX Taq and Rox Reference Dye II (Takara, Japan). The qPCR conditions were as follows: 95 °C 30s, 95 °C 5 s, 60 °C 30s with 40 cycles and finished by Applied Biosystems 7500 Real-Time PCR System (ABI, America). Relative gene expression level was calculated by 2^-ΔΔCT^. NADPH was used as a reference gene. All the primers were listed in Sup Table 2.

## Supplementary Information

**Additional file 1: Sup Fig. 1.** PCA of transcriptome sequencing. The X21CK.V23, X22CK.V23, and X23CK.V23 represent three biological replications of V23 at 0 h cold stress. X1.1 CK VH1, X1.2 CK VH2, and X1.3 CK VH3 represent three biological replications of VH3 at 0 h cold stress. The V23.1, V23.2, and V23.3 represent three biological replications of V23 at 40 h cold stress. The VH3.1, VH3.2, and VH3.3 represent three biological replications of VH3 at 4 h cold stress. (A) X and Y axis indicate PC1 and PC2, respectively. (B) X and Y axis indicate PC1 and PC3, respectively. **Sup Fig. 2.** GA3 contents in V23 and VH3. Data shown re average ± standard error with three replications. ** *P* < 0.01 (Students' t-test). **Sup Table1.** The gene gene IDs and names of DEGs. **Sup Table 2.** Primers used for qRT-PCR.

## Data Availability

All datasets of transcriptome sequencing analyzed during this study are available in NCBI under the accession NO. SAMN12638446, SAMN12638447, SAMN12638448, SAMN12638449, SAMN12638450, SAMN12638451, SAMN12638452, SAMN12638453, SAMN12638454, SAMN12638455, SAMN12638456, and SAMN12638457 (http://www.ncbi.nlm.nih.gov/bioproject/562321). The associated BioProject is PRJNA562321.

## Abbreviations

*V. volvacea*: *Volvariella volvacea*
DEG: Differentially expressed gene
CST: Cold stress trreatment
ROS: Reactive oxygen species
MDA: Malondialdehyde
SOD: Superoxide dismutase
CAT: Catalase
KEGG: Kyoto encyclopedia of genes and genome
GO: Gene ontology
ABA: Abscisic acid
GA: Gibberellic acid
