# Supplementary material for: The cold - resistance mechanism of a mutagenic Volvariella volvacea strain VH3 with outstanding traits revealed by transcriptome profiling
Source: BMC Microbiol. 2021 Dec 8;21:336. doi: 10.1186/s12866-021-02396-8 (PMC8653554; doi:10.1186/s12866-021-02396-8)
Supplement: Supplementary file 1 — Additional file 1: Sup Fig. 1. PCA of transcriptome sequencing. The X21CK.V23, X22CK.V23, and X23CK.V23 represent three biological replications of V23 at 0 h cold stress. X1.1 CK VH1, X1.2 CK VH2, and X1.3 CK VH3 represent three biological replications of VH3 at 0 h cold stress. The V23.1, V23.2, and V23.3 represent three biological replications of V23 at 40 h cold stress. The VH3.1, VH3.2, and VH3.3 represent three biological replications of VH3 at 4 h cold stress. (A) X and Y axis indicate PC1 and PC2, respectively. (B) X and Y axis indicate PC1 and PC3, respectively. Sup Fig. 2. GA3 contents in V23 and VH3. Data shown re average ± standard error with three replications. ** P < 0.01 (Students' t-test). Sup Table1. The gene gene IDs and names of DEGs. Sup Table 2. Primers used for qRT-PCR. [file 12866_2021_2396_MOESM1_ESM.docx]

Supplementary file

Sup Fig.1


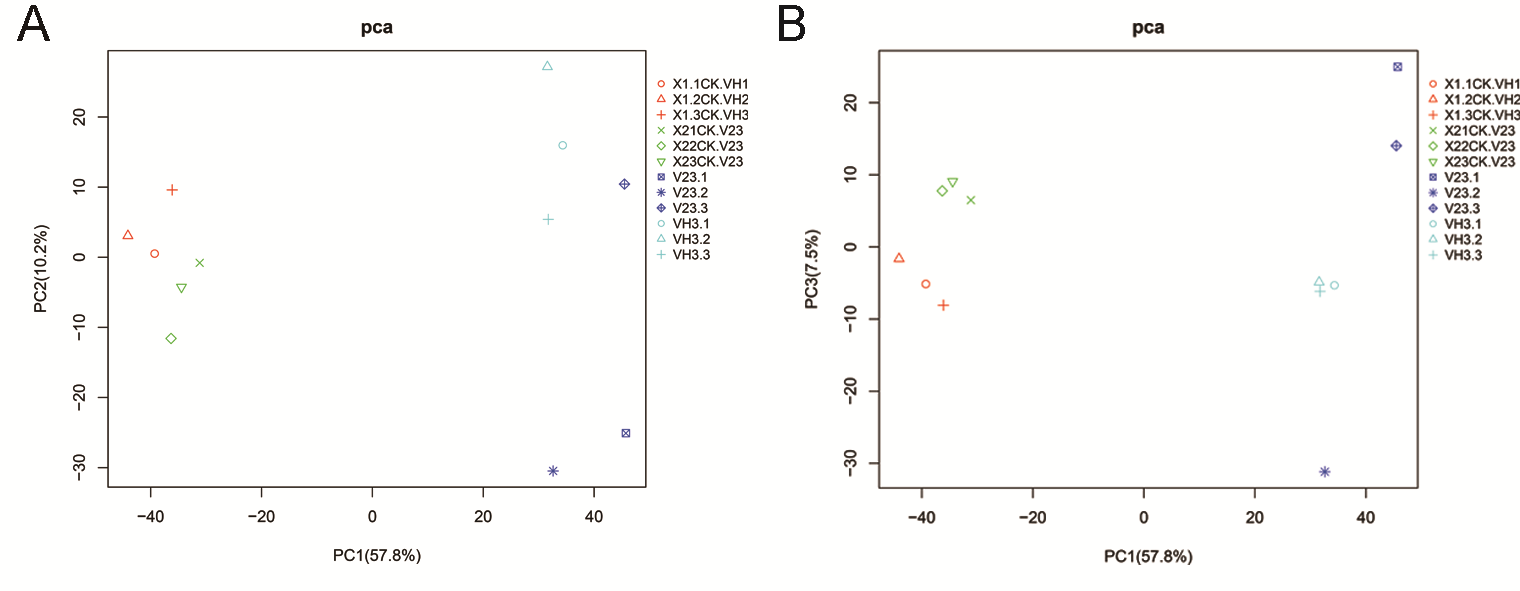


Sup Fig.1. PCA of transcriptome sequencing. The X21CK.V23, X22CK.V23, and X23CK.V23 represent three biological replications of V23 at 0h cold stress. X1.1 CK VH1, X1.2 CK VH2, and X1.3 CK VH3 represent three biological replications of VH3 at 0h cold stress. The V23.1, V23.2, and V23.3 represent three biological replications of V23 at 40h cold stress. The VH3.1, VH3.2, and VH3.3 represent three biological replications of VH3 at 4h cold stress. (A) X and Y axis indicate PC1 and PC2, respectively. (B) X and Y axis indicate PC1 and PC3, respectively.

Sup Fig. 2


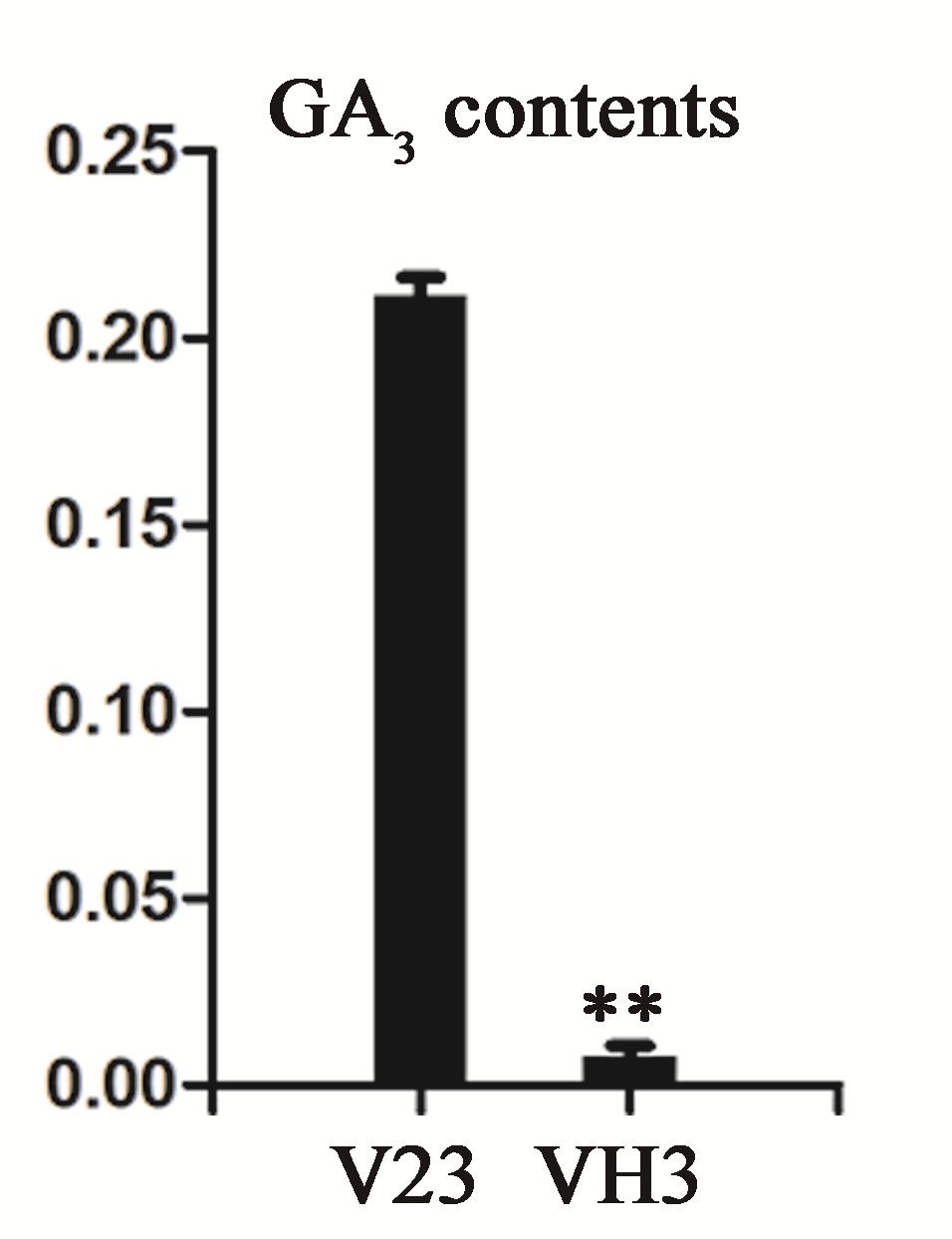


Sup Fig. 2 GA3 contents in V23 and VH3. Data shown re average ± standard error with three replications. ** P < 0.01(Students' *t*-test).

Sup table1

The gene IDs and names of DEGs

| gene ID | gene name | up/down | comparison |
| --- | --- | --- | --- |
| 117932 | VVO-00001 | DOWN | V23-0_vs_VH3-0 |
| 111707 | VVO-00002 | DOWN | V23-0_vs_VH3-0 |
| 114610 | VVO-00003 | DOWN | V23-0_vs_VH3-0 |
| 114609 | VVO-00004 | DOWN | V23-0_vs_VH3-0 |
| 120552 | VVO-00005 | DOWN | V23-0_vs_VH3-0 |
| 115354 | VVO-00006 | UP | V23-0_vs_VH3-0 |
| 120997 | VVO-00007 | DOWN | V23-0_vs_VH3-0 |
| 115930 | VVO-00008 | DOWN | V23-0_vs_VH3-0 |
| 117456 | VVO-00009 | DOWN | V23-0_vs_VH3-0 |
| 117011 | VVO-00010 | DOWN | V23-0_vs_VH3-0 |
| 121881 | VVO-00011 | UP | V23-0_vs_VH3-0 |
| 116442 | VVO-00012 | DOWN | V23-0_vs_VH3-0 |
| 119897 | VVO-00013 | DOWN | V23-0_vs_VH3-0 |
| 116862 | VVO-00014 | UP | V23-0_vs_VH3-0 |
| 116991 | VVO-00015 | DOWN | V23-0_vs_VH3-0 |
| 113719 | VVO-00016 | DOWN | V23-0_vs_VH3-0 |
| 118636 | VVO-00017 | UP | V23-0_vs_VH3-0 |
| 116107 | VVO-00018 | DOWN | V23-0_vs_VH3-0 |
| 115175 | VVO-00019 | UP | V23-0_vs_VH3-0 |
| 117507 | VVO-00020 | UP | V23-0_vs_VH3-0 |
| 119569 | VVO-00021 | UP | V23-0_vs_VH3-0 |
| 120160 | VVO-00022 | DOWN | V23-0_vs_VH3-0 |
| 113712 | VVO-00023 | DOWN | V23-0_vs_VH3-0 |
| 116992 | VVO-00024 | DOWN | V23-0_vs_VH3-0 |
| 115762 | VVO-00025 | UP | V23-0_vs_VH3-0 |
| 111565 | VVO-00026 | UP | V23-0_vs_VH3-0 |
| 115850 | VVO-00027 | DOWN | V23-0_vs_VH3-0 |
| 111608 | VVO-00028 | DOWN | V23-0_vs_VH3-0 |
| 116036 | VVO-00029 | UP | V23-0_vs_VH3-0 |
| 120839 | VVO-00030 | DOWN | V23-0_vs_VH3-0 |
| 119475 | VVO-00031 | UP | V23-0_vs_VH3-0 |
| 122015 | VVO-00032 | DOWN | V23-0_vs_VH3-0 |
| 113664 | VVO-00033 | DOWN | V23-0_vs_VH3-0 |
| 117479 | VVO-00034 | UP | V23-0_vs_VH3-0 |
| 117855 | VVO-00035 | DOWN | V23-0_vs_VH3-0 |
| 115619 | VVO-00036 | UP | V23-0_vs_VH3-0 |
| 112820 | VVO-00037 | DOWN | V23-0_vs_VH3-0 |
| 113354 | VVO-00038 | DOWN | V23-0_vs_VH3-0 |
| 121279 | VVO-00039 | UP | V23-0_vs_VH3-0 |
| 117370 | VVO-00040 | DOWN | V23-0_vs_VH3-0 |
| 116575 | VVO-00041 | DOWN | V23-0_vs_VH3-0 |
| 114064 | VVO-00042 | DOWN | V23-0_vs_VH3-0 |
| 120411 | VVO-00043 | DOWN | V23-0_vs_VH3-0 |
| 118990 | VVO-00044 | DOWN | V23-0_vs_VH3-0 |
| 116135 | VVO-00045 | DOWN | V23-0_vs_VH3-0 |
| 116385 | VVO-00046 | UP | V23-0_vs_VH3-0 |
| 120710 | VVO-00047 | DOWN | V23-0_vs_VH3-0 |
| 111664 | VVO-00048 | UP | V23-0_vs_VH3-0 |
| 119159 | VVO-00049 | DOWN | V23-0_vs_VH3-0 |
| 119365 | VVO-00050 | UP | V23-0_vs_VH3-0 |
| 121606 | VVO-00051 | UP | V23-0_vs_VH3-0 |
| 115195 | VVO-00052 | DOWN | V23-0_vs_VH3-0 |
| 121845 | VVO-00053 | UP | V23-0_vs_VH3-0 |
| 119611 | VVO-00054 | UP | V23-0_vs_VH3-0 |
| 118472 | VVO-00055 | DOWN | V23-0_vs_VH3-0 |
| 114038 | VVO-00056 | UP | V23-0_vs_VH3-0 |
| 112736 | VVO-00057 | UP | V23-0_vs_VH3-0 |
| 114028 | VVO-00058 | UP | V23-0_vs_VH3-0 |
| 116899 | VVO-00059 | UP | V23-0_vs_VH3-0 |
| 114059 | VVO-00060 | DOWN | V23-0_vs_VH3-0 |
| 119842 | VVO-00061 | UP | V23-0_vs_VH3-0 |
| 116870 | VVO-00062 | UP | V23-0_vs_VH3-0 |
| 115213 | VVO-00063 | DOWN | V23-0_vs_VH3-0 |
| 118683 | VVO-00064 | DOWN | V23-0_vs_VH3-0 |
| 118773 | VVO-00065 | UP | V23-0_vs_VH3-0 |
| 113921 | VVO-00066 | UP | V23-0_vs_VH3-0 |
| 116307 | VVO-00067 | UP | V23-0_vs_VH3-0 |
| 115613 | VVO-00068 | DOWN | V23-0_vs_VH3-0 |
| 115067 | VVO-00069 | UP | V23-0_vs_VH3-0 |
| 116654 | VVO-00070 | UP | V23-0_vs_VH3-0 |
| 119109 | VVO-00071 | UP | V23-0_vs_VH3-0 |
| 119588 | VVO-00072 | UP | V23-0_vs_VH3-0 |
| 119714 | VVO-00073 | UP | V23-0_vs_VH3-0 |
| 114259 | VVO-00074 | UP | V23-0_vs_VH3-0 |
| 115740 | VVO-00075 | UP | V23-0_vs_VH3-0 |
| 118038 | VVO-00076 | UP | V23-0_vs_VH3-0 |
| 121694 | VVO-00077 | UP | V23-0_vs_VH3-0 |
| 121558 | VVO-00078 | UP | V23-0_vs_VH3-0 |
| 120076 | VVO-00079 | DOWN | V23-0_vs_VH3-0 |
| 121706 | VVO-00080 | UP | V23-0_vs_VH3-0 |
| 112263 | VVO-00081 | UP | V23-0_vs_VH3-0 |
| 117273 | VVO-00082 | UP | V23-0_vs_VH3-0 |
| 120168 | VVO-00083 | UP | V23-0_vs_VH3-0 |
| 121072 | VVO-00084 | UP | V23-0_vs_VH3-0 |
| 118245 | VVO-00085 | UP | V23-0_vs_VH3-0 |
| 115334 | VVO-00086 | UP | V23-0_vs_VH3-0 |
| 117020 | VVO-00087 | DOWN | V23-0_vs_VH3-0 |
| 119366 | VVO-00088 | UP | V23-0_vs_VH3-0 |
| 119577 | VVO-00089 | UP | V23-0_vs_VH3-0 |
| 115625 | VVO-00090 | DOWN | V23-0_vs_VH3-0 |
| 117264 | VVO-00091 | UP | V23-0_vs_VH3-0 |
| 113764 | VVO-00092 | UP | V23-0_vs_VH3-0 |
| 120109 | VVO-00093 | UP | V23-0_vs_VH3-0 |
| 120178 | VVO-00094 | DOWN | V23-0_vs_VH3-0 |
| 121024 | VVO-00095 | DOWN | V23-0_vs_VH3-0 |
| 111292 | VVO-00096 | DOWN | V23-0_vs_VH3-0 |
| 120129 | VVO-00097 | UP | V23-0_vs_VH3-0 |
| 120767 | VVO-00098 | UP | V23-0_vs_VH3-0 |
| 118463 | VVO-00099 | UP | V23-0_vs_VH3-0 |
| 119788 | VVO-00100 | UP | V23-0_vs_VH3-0 |
| 120893 | VVO-00101 | DOWN | V23-0_vs_VH3-0 |
| 116355 | VVO-00102 | UP | V23-0_vs_VH3-0 |
| 121761 | VVO-00103 | UP | V23-0_vs_VH3-0 |
| 112219 | VVO-00104 | UP | V23-0_vs_VH3-0 |
| 114855 | VVO-00105 | UP | V23-0_vs_VH3-0 |
| 121192 | VVO-00106 | DOWN | V23-0_vs_VH3-0 |
| 118749 | VVO-00107 | DOWN | V23-0_vs_VH3-0 |
| 118780 | VVO-00108 | UP | V23-0_vs_VH3-0 |
| 117552 | VVO-00109 | UP | V23-0_vs_VH3-0 |
| 121438 | VVO-00110 | DOWN | V23-0_vs_VH3-0 |
| 111165 | VVO-00111 | DOWN | V23-0_vs_VH3-0 |
| 121216 | VVO-00112 | UP | V23-4_vs_VH3-4 |
| 114610 | VVO-00003 | DOWN | V23-4_vs_VH3-4 |
| 114609 | VVO-00004 | DOWN | V23-4_vs_VH3-4 |
| 117840 | VVO-00113 | UP | V23-4_vs_VH3-4 |
| 121289 | VVO-00114 | UP | V23-4_vs_VH3-4 |
| 121106 | VVO-00115 | DOWN | V23-4_vs_VH3-4 |
| 112286 | VVO-00116 | UP | V23-4_vs_VH3-4 |
| 118031 | VVO-00117 | UP | V23-4_vs_VH3-4 |
| 119569 | VVO_00021 | UP | V23-4_vs_VH3-4 |
| 112258 | VVO-00118 | UP | V23-4_vs_VH3-4 |
| 122077 | VVO-00119 | UP | V23-4_vs_VH3-4 |
| 114254 | VVO-00120 | UP | V23-4_vs_VH3-4 |
| 116081 | VVO-00121 | DOWN | V23-4_vs_VH3-4 |
| 119302 | VVO-00122 | UP | V23-4_vs_VH3-4 |
| 112249 | VVO-00123 | UP | V23-4_vs_VH3-4 |
| 119816 | VVO-00124 | UP | V23-4_vs_VH3-4 |
| 114333 | VVO-00125 | DOWN | V23-4_vs_VH3-4 |
| 114849 | VVO-00126 | UP | V23-4_vs_VH3-4 |
| 119808 | VVO-00127 | UP | V23-4_vs_VH3-4 |
| 119875 | VVO-00128 | UP | V23-4_vs_VH3-4 |
| 121211 | VVO-00129 | UP | V23-4_vs_VH3-4 |
| 115769 | VVO-00130 | UP | V23-4_vs_VH3-4 |
| 115306 | VVO-00131 | DOWN | V23-4_vs_VH3-4 |
| 116776 | VVO-00132 | DOWN | V23-4_vs_VH3-4 |
| 117598 | VVO-00133 | UP | V23-4_vs_VH3-4 |
| 117842 | VVO-00134 | DOWN | V23-4_vs_VH3-4 |
| 118093 | VVO-00135 | UP | V23-4_vs_VH3-4 |
| 115975 | VVO-00136 | DOWN | V23-4_vs_VH3-4 |
| 119826 | VVO-00137 | UP | V23-4_vs_VH3-4 |
| 114029 | VVO-00138 | UP | V23-4_vs_VH3-4 |
| 115127 | VVO-00139 | UP | V23-4_vs_VH3-4 |
| 112620 | VVO-00140 | DOWN | V23-4_vs_VH3-4 |
| 117292 | VVO-00141 | UP | V23-4_vs_VH3-4 |
| 119811 | VVO-00142 | UP | V23-4_vs_VH3-4 |
| 120112 | VVO-00143 | UP | V23-4_vs_VH3-4 |
| 113376 | VVO-00144 | UP | V23-4_vs_VH3-4 |
| 111637 | VVO-00145 | UP | V23-4_vs_VH3-4 |
| 119810 | VVO-00146 | UP | V23-4_vs_VH3-4 |
| 118597 | VVO-00147 | UP | V23-4_vs_VH3-4 |
| 117585 | VVO-00148 | UP | V23-4_vs_VH3-4 |
| 113613 | VVO-00149 | UP | V23-4_vs_VH3-4 |
| 115079 | VVO-00150 | DOWN | V23-4_vs_VH3-4 |
| 114133 | VVO-00151 | UP | V23-4_vs_VH3-4 |
| 121231 | VVO-00152 | UP | V23-4_vs_VH3-4 |
| 120427 | VVO-00153 | UP | V23-4_vs_VH3-4 |
| 120854 | VVO-00154 | UP | V23-4_vs_VH3-4 |
| 116274 | VVO-00155 | UP | V23-4_vs_VH3-4 |
| 116814 | VVO-00156 | UP | V23-4_vs_VH3-4 |
| 116022 | VVO-00157 | UP | V23-4_vs_VH3-4 |
| 120562 | VVO-00158 | UP | V23-4_vs_VH3-4 |
| 121607 | VVO-00159 | UP | V23-4_vs_VH3-4 |
| 115666 | VVO-00160 | UP | V23-4_vs_VH3-4 |
| 117512 | VVO-00161 | UP | V23-4_vs_VH3-4 |
| 118536 | VVO-00162 | UP | V23-4_vs_VH3-4 |
| 119536 | VVO-00163 | UP | V23-4_vs_VH3-4 |
| 121855 | VVO-00164 | UP | V23-4_vs_VH3-4 |
| 116024 | VVO-00165 | UP | V23-4_vs_VH3-4 |
| 119756 | VVO-00166 | UP | V23-4_vs_VH3-4 |
| 112251 | VVO-00167 | UP | V23-4_vs_VH3-4 |
| 115507 | VVO-00168 | UP | V23-4_vs_VH3-4 |
| 119721 | VVO-00169 | UP | V23-4_vs_VH3-4 |
| 121582 | VVO-00170 | UP | V23-4_vs_VH3-4 |
| 119400 | VVO-00171 | UP | V23-4_vs_VH3-4 |
| 114564 | VVO-00172 | UP | V23-4_vs_VH3-4 |
| 115447 | VVO-00173 | UP | V23-4_vs_VH3-4 |
| 119487 | VVO-00174 | UP | V23-4_vs_VH3-4 |
| 120888 | VVO-00175 | UP | V23-4_vs_VH3-4 |
| 118862 | VVO-00176 | UP | V23-4_vs_VH3-4 |
| 111626 | VVO-00177 | UP | V23-4_vs_VH3-4 |
| 118163 | VVO-00178 | UP | V23-4_vs_VH3-4 |
| 120172 | VVO-00179 | UP | V23-4_vs_VH3-4 |
| 120665 | VVO-00180 | DOWN | V23-4_vs_VH3-4 |
| 113694 | VVO-00181 | UP | V23-4_vs_VH3-4 |
| 117397 | VVO-00182 | UP | V23-4_vs_VH3-4 |
| 118974 | VVO-00183 | UP | V23-4_vs_VH3-4 |
| 115307 | VVO-00184 | DOWN | V23-4_vs_VH3-4 |
| 116872 | VVO-00185 | UP | V23-4_vs_VH3-4 |
| 111653 | VVO-00186 | DOWN | V23-4_vs_VH3-4 |
| 114350 | VVO-00187 | UP | V23-4_vs_VH3-4 |
| 120839 | VVO-00030 | DOWN | V23-4_vs_VH3-4 |
| 112402 | VVO-00188 | UP | V23-4_vs_VH3-4 |
| 119127 | VVO-00189 | UP | V23-4_vs_VH3-4 |
| 114700 | VVO-00190 | UP | V23-4_vs_VH3-4 |
| 112639 | VVO-00191 | UP | V23-4_vs_VH3-4 |
| 120360 | VVO-00192 | UP | V23-4_vs_VH3-4 |
| 118865 | VVO-00193 | UP | V23-4_vs_VH3-4 |
| 111064 | VVO-00194 | UP | V23-4_vs_VH3-4 |
| 117124 | VVO-00195 | DOWN | V23-4_vs_VH3-4 |
| 118319 | VVO-00196 | UP | V23-4_vs_VH3-4 |
| 115113 | VVO-00197 | UP | V23-4_vs_VH3-4 |
| 115287 | VVO-00198 | UP | V23-4_vs_VH3-4 |
| 116371 | VVO-00199 | UP | V23-4_vs_VH3-4 |
| 118663 | VVO-00200 | UP | V23-4_vs_VH3-4 |
| 113390 | VVO-00201 | UP | V23-4_vs_VH3-4 |
| 111928 | VVO-00202 | UP | V23-4_vs_VH3-4 |
| 114139 | VVO-00203 | DOWN | V23-4_vs_VH3-4 |
| 117985 | VVO-00204 | UP | V23-4_vs_VH3-4 |
| 122007 | VVO-00205 | UP | V23-4_vs_VH3-4 |
| 111683 | VVO-00206 | DOWN | V23-4_vs_VH3-4 |
| 121861 | VVO-00207 | UP | V23-4_vs_VH3-4 |
| 118636 | VVO-00017 | UP | V23-4_vs_VH3-4 |
| 119170 | VVO-00208 | UP | V23-4_vs_VH3-4 |
| 119479 | VVO-00209 | UP | V23-4_vs_VH3-4 |
| 114547 | VVO-00210 | UP | V23-4_vs_VH3-4 |
| 114983 | VVO-00211 | UP | V23-4_vs_VH3-4 |
| 121565 | VVO-00212 | DOWN | V23-4_vs_VH3-4 |
| 112294 | VVO-00213 | UP | V23-4_vs_VH3-4 |
| 112325 | VVO-00214 | UP | V23-4_vs_VH3-4 |
| 121108 | VVO-00215 | UP | V23-4_vs_VH3-4 |
| 113961 | VVO-00216 | DOWN | V23-4_vs_VH3-4 |
| 117011 | VVO-00010 | DOWN | V23-4_vs_VH3-4 |
| 114895 | VVO-00217 | UP | V23-4_vs_VH3-4 |
| 118818 | VVO-00218 | DOWN | V23-4_vs_VH3-4 |
| 115441 | VVO-00219 | UP | V23-4_vs_VH3-4 |
| 119435 | VVO-00220 | UP | V23-4_vs_VH3-4 |
| 120117 | VVO-00221 | DOWN | V23-4_vs_VH3-4 |
| 114080 | VVO-00222 | UP | V23-4_vs_VH3-4 |
| 115698 | VVO-00223 | UP | V23-0_vs_V23-4 |

Sup table 2

Primers used for qRT-PCR

| Gene name | Forward primer | Reverse primer |
| --- | --- | --- |
| NADPH (reference gene) | attggcgtggtggtcgtag | acggaaacatcaagggtaggg |
| VVO_00048 | ccgttccaactggttactgg | gcccttcacaccaccaaa |
| VVO_00087 | gccacctcttctccgaatc | acctgtccggactcgatg |
| VVO_00120 | gcattgagcttggaaggaagt | cttcttcggcacgcttattc |
| VVO_00164 | aggtgtatttgtgctacagattgaa | tatccgcctctccggatt |
| VVO_00219 | cgtcgaaacgatacggaaat | tccttgaaccacggatgtg |
| VVO_00223 | atggccgcaacttgtctg | cgctgctcttgctaatctcc |
